# Somatic cell count and alkaline phosphatase activity in milk for evaluation of mastitis in buffalo

**DOI:** 10.14202/vetworld.2015.363-366

**Published:** 2015-03-21

**Authors:** M. P. Patil, A. S. Nagvekar, S. D. Ingole, S. V. Bharucha, V. T. Palve

**Affiliations:** 1 Department of Veterinary Physiology, College of Animal Husbandry, Sharadanagar, Baramati, Pune, Maharashtra, India; 2 Department of Veterinary Physiology, Bombay Veterinary College, Maharashtra Animal and Fishery Sciences University, Parel, Mumbai, Maharashtra, India

**Keywords:** alkaline phosphatase, buffalo milk, mastitis, somatic cell count

## Abstract

**Background and Aim:**

Mastitis is a serious disease of dairy animals causing great economic losses due to a reduction in milk yield as well as lowering its nutritive value. The application of somatic cell count (SCC) and alkaline phosphatase activity in the milk for diagnosis of mastitis in buffalo is not well documented. Therefore, the present study was conducted to observe the SCC and alkaline phosphatase activity for evaluation of mastitis in buffalo.

**Materials and Methods:**

Milk samples of forty apparently healthy lactating buffaloes were selected and categorized into five different groups *viz*. normal buffaloes, buffaloes with subclinical mastitis with CMT positive milk samples (+1 Grade), (+2 Grade), (+3 Grade), and buffaloes with clinical mastitis with 8 animals in each group. The milk samples were analyzed for SCC and alkaline phosphatase activity.

**Results:**

The levels of SCC (×10^5^ cells/ml) and alkaline phosphatase (U/L) in different groups were *viz*. normal (3.21±0.179, 16.48±1.432), subclinical mastitis with CMT positive milk samples with +1 Grade (4.21±0.138, 28.11±1.013), with +2 Grade (6.34±0.183, 34.50±1.034), with +3 Grade (7.96±0.213, 37.73±0.737) and buffaloes with clinical mastitis (10.21±0.220, 42.37±0.907) respectively, indicating an increasing trend in the values and the difference observed among various group was statistically significant.

**Conclusion:**

In conclusion, the results of the present study indicate that the concentration of milk SCC and alkaline phosphatase activity was higher in the milk of buffaloes with mastitis than in the milk of normal buffaloes.

## Introduction

Mastitis is a serious disease of dairy animals causing great economic losses due to a reduction in milk yield as well as lowering its nutritive value. Mastitis is characterized by physical, chemical, and bacteriological changes in the milk and pathological changes in the glandular tissue of the udder [1]. Mastitis affects the milk quality in terms of the composition of the milk and yield of the milk. The extent of various changes in composition depends on the inflammatory response [2]. Somatic cells are mainly milk-secreting epithelial cells that have been shed from the lining of the gland and white blood cells (leukocytes) that have entered the mammary gland in response to injury or infection [3]. Alkaline phosphatase is an enzyme that is naturally found in milk. The presence of this enzyme in the milk at levels elevated above normal suggests an increase rate of tissue destruction. The present study was taken up because the work on somatic cell count (SCC) and alkaline phosphatase activity in milk of normal and mastitic buffalo is not well documented.

Somatic cells are indicators of both resistance and susceptibility of cows to mastitis and can be used to monitor the level or occurrence of subclinical mastitis in herds or individual cows. SCC is a useful predictor of intramammary infection (IMI) and, therefore, an important component of milk in assessment of aspects of quality, hygiene, and mastitis control. The milk somatic cells include 75% leucocytes, i.e. neutrophils, macrophages, lymphocytes, and 25% epithelial cells. During inflammation (mastitis) the major increase in SCC is due to the influx of neutrophils into the milk to fight infection and have been estimated over 90% [4,5] and the measurement of somatic cell in milk is known as a SCC. It is [6] reported that an increase in alkaline phosphatase level in sheep with mastitis may be linked with tissue damage occurring in mammary tissue. A change in the level of this enzyme in normal and mastitic milk has been studied in cattle [7] and camel [8].

The present study was conducted to observe the SCC and alkaline phosphatase activity for evaluation of mastitis in buffalo.

## Materials and Methods

Ethical approval: Not necessary.

Milk samples from forty apparently healthy lactating buffaloes were collected from Instructional Livestock Farm Complex, Unit No-3, Bombay Veterinary College, Aarey milk colony and private farms, Aarey milk colony, Goregaon, Mumbai-400065. The experimental animals were aged between 5 and 13 years and were in their 1^st^-6th lactation. On the basis of thorough clinical examination of udder and careful application of California mastitis test (CMT), these experimental animals were screened and categorized in five different groups *viz*. normal buffaloes, buffaloes with subclinical mastitis with CMT positive milk samples (+1 Grade), (+2 Grade), (+3 Grade), and buffaloes with clinical mastitis with 8 animals in each group.

The buffaloes were maintained under uniform and standard conditions of feeding and management. The animals were housed in animal shed with asbestos cement roof, under natural daylight and temperature conditions. The buffaloes were given maintenance ration of 15 kg of para grass and 2.5 kg of concentrate mixture during morning and evening hours daily. Paddy straw was given *ad-libitum*.

After thorough clinical examination of the udder and performing CMT carefully, 40 milk samples were collected. First stream of milk was discarded and then about 10-20 ml of milk was collected aseptically from each teat into clean and dry plastic bottles by hand milking. All these milk samples were collected and kept immediately into ice bath to carry out subsequent physiochemical analysis in the laboratory.

The SCC in milk was done as per the standard method described by Schalm *et al* [9] and the activity of alkaline phosphatase in whey was estimated by using an autoanalyzer- Prietest Touch (Robonik, India).

### Statistical analysis

Statistical analysis of the data was done by complete randomized design as per described by Snedecor and Cochran [10].

## Results

A total of 40 milk samples were apparently collected from healthy lactating buffaloes. These buffaloes were divided into five groups; as normal animals and animals with subclinical mastitis (SCM) (+, ++, +++) and clinical mastitis on the basis of CMT.

SCC (×10^5^ cells/ml) and alkaline phosphatase activity (U/L) of milk from normal buffaloes and the buffaloes affected with subclinical (with different Grades as per CMT) and clinical mastitis are presented in Table-1, the same is depicted graphically in Figure-1 (SCC) and Figure-2 (alkaline phosphatase). From Table-1, it is clear that the mean values of SCC and alkaline phosphatase activity were in ascending order. The SCC (3.21±0.179 × 10^5^ cells/ml) and alkaline phosphatase activity (16.48±1.432 U/L) were lowest in normal milk. However, the SCC (10.21±0.22 × 10^5^ cells/ml) and alkaline phosphatase activity (42.37±0.907 U/L) were highest in clinical mastitic milk. The values of SCC and alkaline phosphatase activity in subclinical mastitis milk group graded with (+, ++, +++) were (4.21±0.138, 6.34±0.183, 7.96±0.213 × 10^5^ cells/ml) and 28.11±1.013, 34.50±1.034, 37.73±0.737 U/L) respectively. The results indicate that the increase of SCC and alkaline phosphatase activity in milk increase with the severity of IMI or mastitis.

**Table-1:** SCC (10^5^ cells/ml) and alkaline phosphatase (U/L) of milk from normal buffaloes and the buffaloes affected with subclinical and clinical mastitis.

Group	SCC×10^5^ cells/ml	Alkaline phosphatase (U/L)
Control	3.21±0.179^a^	16.48±1.432^a^
Subclinical +	4.21±0.138^b^	28.11±1.013^b^
Subclinical ++	6.34±0.183^c^	34.50±1.034^c^
Subclinical +++	7.96±0.213^d^	37.73±0.737^d^
Clinical	10.21±0.220^e^	42.37±0.907^e^

Mean with at least one common superscript do not differ significantly (p<0.05), SCC: Somatic cell count

## Discussion

The SCC was higher in buffaloes with subclinical mastitis than in buffaloes with normal milk. The SCC was higher in buffaloes with clinical mastitis than those with subclinical mastitis (Table-1 and Figure-1). The difference in SCC between normal, subclinical, and clinical mastitis was highly (p<0.05) significant.

The results of the present study are in agreement with previous studies in buffaloes [11-17] and in cows [5,18-22].

Somatic cells are mainly milk-secreting epithelial cells that have been shed from the lining of the gland and white blood cells (leukocytes) that have entered the mammary gland in response to injury or infection. There are plenty of factors like stage of lactation, age, breed, parity, season and stress that influence milk SCC apart from IMI [3]. Harmon [5] reported that a modest rise in the SCC of the uninfected quarters at the end of lactation which is, in fact, a dilution effect. Barlett, *et al*. [23] observed that increased SCC in older cattle and/or at the end of lactation is due to increased prevalence of infection and permanent glandular damage from previous infections. The average cell count of 1.99±0.03 × 10^5^ cells/ml with the range of 1.86 × 10^5^-2.12 × 10^5^ cells/ml of milk were reported from healthy quarters of buffaloes [16]. Kavitha, *et al*. [15] observed that the mean normal SCC was 1, 60, 536 cells/ml of milk. Silva, *et al* [11] reported that total SCC in milk from normal buffaloes varied from 0.5 × 10^5^ to 3.75 × 10^5^ cells/ml. Mean SCC of 3, 21, 000±0.179 cells/ml in milk from normal buffalo is reported in the present study and this value is less than the standard set by European Union Directives (92/46 CEE and 94/71 CEE) of 400000 somatic cells/ml of raw buffalo milk [3].

Hussain, *et al*. [17] reported that the SCC was distinctly greater in infected quarters of buffaloes. Dhakal [12] diagnosed subclinical mastitis on the basis of samples with SCC ≥200000/ml with positive bacterial cultures while Sharif, *et al*. [14] reported that SCC increased with severity of subclinical mastitis. Nandi [16] reported that the average SCC in the sub-clinical +, ++ and +++ were 3.72-4.32 × 10^5^, 5.63-7.01 × 10^5^ and 7.56-8.76 × 10^5^ cells/ml respectively. However, results of the present study are higher than those reported by Kavitha, *et al*. [15] for subclinical mastitis (2.59 lakhs somatic cells/ml of milk). The mean SCC in milk from buffaloes with clinical mastitis in the present study was 10.21±0.22 × 10^5^ cells/ml and it is in agreement with that reported by Nandi [16] (10.13±0.19 × 10^5^ cells/ml) for clinical mastitis, however, the values reported are not in agreement with Kavitha, *et al*. [15] who reported 6.05 lakhs somatic cells/ml of milk from buffalo with clinical mastitis.

All lactating animals have a low baseline SCC even if they do not have an IMI. When an infection is detected by the immune system in a healthy buffalo or cow a rapid influx of leukocytes will quickly raise the SCC far beyond the baseline level, usually to over a million cells/ml. In most developed dairy industries various regulatory limits has been applied to milk for human consumption. Mastitis should be detected in a reliable and timely fashion based on SCC values, otherwise subclinical mastitis could develop into a clinical disease [24].

The alkaline phosphatase activity was higher in buffaloes with subclinical mastitis than in buffaloes with normal milk. The alkaline phosphatase activity was higher in buffaloes with clinical mastitis than those with subclinical mastitis (Table-1 and Figure-2). The difference in concentration of alkaline phosphatase activity between normal, subclinical (+, ++, +++), and clinical mastitis were highly (p<0.05) significant.

The increase in milk alkaline phosphatase concentration in mastitis animals may be linked with tissue damage occurring in mammary tissue [6]. The increased level of ALP in milk occurs mainly due to increase permeability of microcirculatory vessels in inflamed areas along with leakage from degenerated/necrotic parenchyma cells and leukocytes [25]. Reported that the quantity and enzymatic activity of the alkaline phosphatase increased in the mastitic milk; therefore its measurement can constitute an indicator to identify an infectious process in mammary gland.

## Conclusion

The results of the present study indicate that the concentration of milk SCC and alkaline phosphatase activity was higher in buffaloes with mastitis than in the milk of normal buffaloes. The SCC and alkaline phosphatase of milk was higher in clinical mastitis than in subclinical mastitis. Severity of mammary gland inflammation was accompanied by increase in SCC. Changes in alkaline phosphatase activities of milk can be a consequence of cell structural damage and, therefore, alkaline phosphatase activity test may be reliable and can be used along with SCC for early diagnosis of subclinical mastitis.

## Authors’ Contributions

ASN supervised the research. MPP was involved in sample collection and processing of samples in the laboratory. MPP, VTP and ASN participated in draft and revision of the manuscript. SDI and SVB guided and co-operated throughout the research. All authors read and approved the final manuscript.
